# Oxygen therapy for children: A key tool in reducing deaths from pneumonia

**DOI:** 10.1002/ppul.24656

**Published:** 2020-01-21

**Authors:** Hamish Graham, Ayobami A. Bakare, Chizoba Fashanu, Owens Wiwa, Trevor Duke, Adegoke G. Falade

**Affiliations:** ^1^ Centre for International Child Health, Royal Children's Hospital, MCRI University of Melbourne Parkville Australia; ^2^ Department of Paediatrics University College Hospital Ibadan Nigeria; ^3^ Department of Community Medicine University College Hospital Ibadan Nigeria; ^4^ Clinton Health Access Initiative Abuja Nigeria; ^5^ Department of Paediatrics University of Ibadan Ibadan Nigeria

## 1 INTRODUCTION

Oxygen is one of the most basic medical therapies we have for acute respiratory illnesses and it has been an established part of medical practice for over 100 years. However, most patients who may benefit from oxygen in low‐ and middle‐income countries will not receive it—either because oxygen is not available or because their need for oxygen is unrecognized. This gap takes an enormous toll, with modeling estimates suggesting that improved pulse oximetry and oxygen access could avert 148 000 under‐five child pneumonia deaths annually in the 15 countries with the highest pneumonia burden^1^. This commentary explores the reasons for this gap, using Nigeria as an illustrative case study in how oxygen access can be improved globally, and concluding with key actions for policy and practice.

## 2 CHILD MORTALITY AND HYPOXEMIA IN NIGERIA

Nigeria is a large lower middle‐income country that ranks second in the number of child pneumonia deaths globally, contributing one‐sixth of under‐five pneumonia deaths globally.^1^, ^2^ Pneumonia causes 18% of under‐five deaths in Nigeria, followed by malaria (14%), complications of prematurity (12%), neonatal encephalopathy and trauma (11%), and diarrheal diseases (10%).^2^ Nigerian studies suggest that hypoxemia affects approximately 14% of children admitted to hospital, including 28% to 49% of children with pneumonia and 22% to 41% of neonates.^3^, ^4^, ^5^, ^6^ Hypoxemia ranks alongside severe acute malnutrition as a major predictor of mortality, with recent data from 12 Nigerian hospitals showing that children with hypoxemia had seven‐fold higher risk of death than those who did not.^3^

Despite the high burden of hypoxemia and increasing recognition of its position as a key sign of illness severity, pulse oximetry, and oxygen therapy have been inadequately used in most Nigerian hospitals. Facility evaluations of Nigerian hospitals have shown that oxygen supply is often not available on pediatric wards, pulse oximeters are uncommonly used outside operating theaters, and healthcare workers lack training on how to use oxygen and pulse oximeters.^7^, ^8^

These findings in Nigeria are echoed globally. Hypoxemia is common and deadly,^9^, ^10^ yet access to oxygen and pulse oximeters remains limited and healthcare worker skills and confidence using oxygen is low^11^, ^12^, ^13^, ^14^—especially in health centers and smaller hospitals where most sick children first present.

## 3 KEY BARRIERS TO IMPROVING OXYGEN ACCESS AND USE

At the hospital level, there are three key barriers to improving oxygen access and use.

### 3.1 First, oxygen‐related equipment, if available, is often low quality, faulty, and poorly maintained

A recent study in 12 south‐west Nigerian hospitals found that 92% (11/12) of hospitals had some access to oxygen supplies, 42% (5/12) had oxygen available on pediatric wards at the time of evaluation, and 8% (1/12) used pulse oximetry for pediatric care.^7^ Testing of 57 oxygen concentrators revealed that 5% (3/57) were producing medical‐grade oxygen (defined as >85% purity), and 48% (24/50) of those that turned on and blew gas were simply blowing out air.^7^ Data from hospitals in northern Nigeria showed that the situation was even worse in these poorer, more rural, locations—11% of hospital pediatric wards had functional oxygen, 2% had pulse oximeters.^8^ In both regions, procurement of oxygen equipment was haphazard, motivated by price and availability of donated items, with little regard to quality or appropriateness. Hospitals lacked preventive maintenance programs and many technicians reported that they were not aware of the procurement of oxygen equipment until it was brought to them broken for repair.^7^

### 3.2 Second, clinical guidance, training, and support to use oxygen is limited and poorly implemented

Oxygen therapy is unlike most other medications, being administered using equipment and titrated by nurses based on serial clinical assessments (including pulse oximetry). Clinical use of oxygen is not complicated, but healthcare workers do require some basic knowledge and skills. In Nigeria, most nurses receive little training on oxygen, and no training on pulse oximetry, either in nursing school or in‐service training—unless they work in anesthetics/theater.^7^ As such, pulse oximetry is rarely used on pediatric wards, and oxygen (when it is available) is used at excessive flow rates and primarily for those with very obvious respiratory distress; many hypoxemic patients will not receive oxygen.^7^, ^8^

### 3.3 Third, oxygen‐related care is expensive to hospitals and patients

Maintaining supply of medical oxygen is expensive, and is made costlier by faulty equipment (eg, leaky cylinders and piping), and poor clinical practices (eg, without pulse oximetry to guide therapy). In Nigeria, oxygen‐related patient fees are substantial, typically around ₦3,900 per day^7^ (USD$21, 2015). These costs fall most heavily on those who are sickest and require longer hospitalization and may result in treatment refusal or discharge against medical advice.

### 3.4 These hospital‐level barriers exist in a social and political context

In Nigeria (and many other countries), health financing deficiencies result in patients facing substantial out‐of‐pocket costs in accessing hospital care. The decentralized hospital system in Nigeria gives individual hospitals more autonomy and responsibility for procuring and maintaining medical equipment. However, without technical support or a strong regulatory framework, hospitals often end up with a haphazard array of cheap equipment that they are unable to maintain or repair—exacerbated further by poorly considered equipment donation programs.

## 4 PROGRESS AND FUTURE OPPORTUNITIES

### 4.1 Progress

Nigerian clinicians, hospital administrators, and policymakers have made substantial progress towards improving oxygen access and use. At the national policy level, the Federal Ministry of Health has revised the essential medicines and equipment lists to include oxygen and oxygen‐related products, updated the pneumonia clinical guidelines, created a new clinical guideline and hospital policy on oxygen,^15^ and developed a national strategy for scaling up oxygen nationally.^16^ This strategy includes the nomination of personnel in the Federal and State Ministries of Health who will be responsible for addressing oxygen access issues in their jurisdiction.

Demonstration projects in Nigeria, have shown that the barriers to improving oxygen services can be overcome using existing commercially available equipment, local maintenance teams, and team‐based approaches to learning and quality improvement. Results from these projects have demonstrated improved pulse oximetry use on pediatric wards (from <20% to >75%), improved oxygen provision to hypoxaemic children (from <20% to >85%), and reduced mortality from child pneumonia.^17^, ^18^

**Figure 1:**
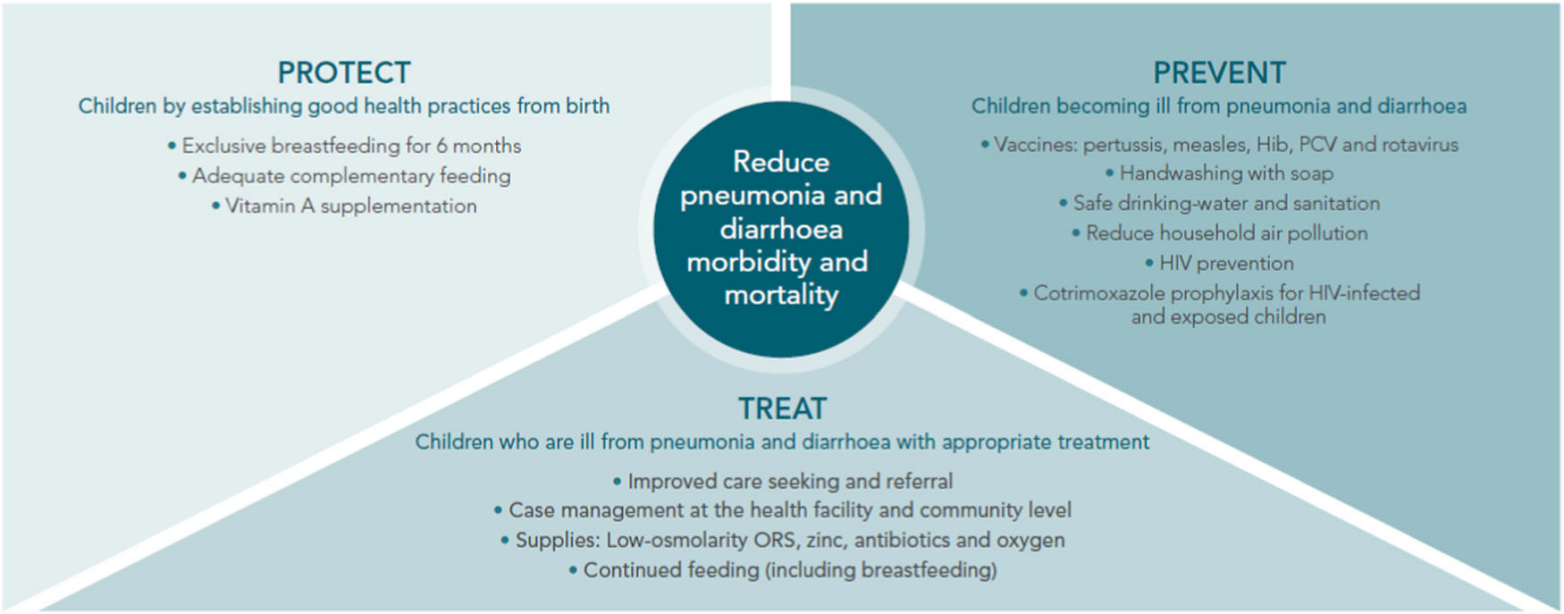
WHO/UNICEF's Protect Prevent Treat framework for reducing mortality from childhood diarrhea and pneumonia, from the Global Action Plan for Pneumonia and Diarrhea (2013)^19^ [Color figure can be viewed at wileyonlinelibrary.com]

Global policies have also shifted to better support oxygen systems strengthening. In 2013, WHO/UNICEF added oxygen to the Global Action Plan for Pneumonia and Diarrhea, correcting an omission from the first edition.^19^, ^20^ This establishes oxygen therapy as a core treatment in the *Protect, Prevent, Treat* framework (Figure 1). In the past 4 years, the WHO has revised the Model List of Essential Medicines for Children to include oxygen for the treatment of hypoxemia in children,^21^ published technical specifications for concentrators^22^ and other oxygen therapy devices,^23^ and released a new oxygen clinical guidelines for children^24^—all of which have been used and adapted by Nigeria and other countries.

### 4.2 Future opportunities

Oxygen therapy is an essential medical therapy for hospital care and should be recognized as a cost‐effective investment for improving health care quality and health outcomes. We identify the following key opportunities for improving oxygen therapy for children globally.

First, existing evidence shows that improving oxygen systems is a cost‐effective intervention that improves the quality of health services and health outcomes. Just as healthcare workers understand the value of oxygen as a basic medical therapy, policy‐makers should now be able to recognize that oxygen is a sound financial investment that will make health services better overall. Better oxygen systems should not only improve access to oxygen therapy but also strengthen broader hospital quality of care systems and stimulate the adoption of other essential health technologies as well.

Second, we know enough to enact national‐scale implementation of improved oxygen systems. Experiences from Nigeria, Ethiopia, Papua New Guinea, and elsewhere, have identified contextual challenges and solutions to improving oxygen systems. These solutions will not be situated within vertically structured programming, but by accepting oxygen therapy as a basic hospital service within a universal health coverage agenda. Policymakers and program managers can be guided by policy documents and technical specifications from WHO and UNICEF^22^, ^23^, ^24^ and Every Breath Counts and United for Oxygen consortiums. However, national oxygen strategies must adopt this guidance to local contexts, defining specific responsibilities for those involved in pharmaceutical, medical device, and financing services.

Third, pulse oximetry is a low‐cost, relatively easily implemented, component of oxygen systems. Essential to the identification of hypoxemia, pulse oximetry is also embraced by healthcare workers as a valuable tool in assessing and monitoring sick patients that improves the quality of care more generally. As such, pulse oximetry scale‐up represents “low‐hanging fruit” for improving hospital care, and may also play an important role in facilitating referral from primary care.^14^, ^25^

Fourth, existing oxygen technologies are suboptimal in hot, humid, dusty conditions, or environments that lack strong maintenance structures. We need better technology to provide reliable oxygen in places where power failures are common, produce and store oxygen locally, and efficiently deliver oxygen from the oxygen source to patients.

Fifth, previous studies have demonstrated the mortality impact of pulse oximetry and improved oxygen systems for young children with pneumonia, but little data exist for other children or neonates (in whom oxygen is also used commonly). The neonatal cohort represents a particular group of interest, as they can suffer adverse effects from administration of excessive oxygen (eg, retinopathy of prematurity, bronchopulmonary dysplasia).^26^, ^27^

## 5 CONCLUSIONS

Oxygen therapy is an essential medical therapy that is poorly available and suboptimally used in many low‐ and middle‐income countries. Recent policy and programmatic experience in Nigeria has shown how oxygen services can be improved for the benefit of children and health services.

## AUTHOR CONTRIBUTIONS

HG drafted the manuscript. AAB, CF, OQ, TD, and AGF provided substantial comments to the writing of the manuscript. All authors read and approved the final manuscript.
